# The Mycobiota of the Deep Sea: What Omics Can Offer

**DOI:** 10.3390/life10110292

**Published:** 2020-11-19

**Authors:** Lluvia Vargas-Gastélum, Meritxell Riquelme

**Affiliations:** Department of Microbiology, Centro de Investigación Científica y Educación Superior de Ensenada (CICESE), Ctra.Ensenada-Tijuana No. 3918, Ensenada 22860, Baja California, Mexico; lvargas@cigom.org

**Keywords:** fungi, deep sea, sediments, water, omics

## Abstract

The deep sea (>1000 m below sea level) represents one of the most extreme environments of the ocean. Despite exhibiting harsh abiotic conditions such as low temperatures, high hydrostatic pressure, high salinity concentrations, a low input of organic matter, and absence of light, the deep sea encompasses a great fungal diversity. For decades, most knowledge on the fungal diversity of the deep sea was obtained through culture-dependent techniques. More recently, with the latest advances of high-throughput next generation sequencing platforms, there has been a rapid increment in the number of studies using culture-independent techniques. This review brings into the spotlight the progress of the techniques used to assess the diversity and ecological role of the deep-sea mycobiota and provides an overview on how the omics technologies have contributed to gaining knowledge about fungi and their activity in poorly explored marine environments. Finally, current challenges and suggested coordinated efforts to overcome them are discussed.

## 1. Introduction

It has been estimated that terrestrial fungi represent ≈2.18% (≈12 Gigatons of carbon; GtC) of the total biomass on Earth (≈550 GtC), while marine fungi represent only ≈0.054% (≈0.03 GtC) [1]. This assessment of marine fungal biomass only takes into account the fungi associated with the planktonic community from the epipelagic to mesopelagic layers of the water column, and the fungi in the deep sea were not considered for that assessment.

The deep-sea biosphere covers more than 65% of the Earth’s surface and comprises the environments located at least 1000 m below sea level (mbsl) [2]. The current knowledge on fungi in the marine environment, particularly in the deep-sea biosphere, is scarce compared to what is known for the terrestrial environment. Fungi detected in the deep-sea range from 1000 m to more than 10,800 mbsl. The latter represents some of the deepest zones in the ocean including the Mariana Trench in the Western Pacific Ocean [3,4]. A lot of work remains to be done to better characterize the fungi of the deep-sea environment and to determine their participation in the different ecological processes.

The aim of this review is to summarize and contrast available information about fungi of the deep sea including the sampled locations, the sampling and characterization tools employed, and the fungal diversity described. We also analyze how the development of omics technologies contributes to the discovery of novel marine fungal lineages and to improve our understanding on the diversity, evolution, ecological role, and biotechnological potential of deep-sea fungi. Finally, we discuss the current challenges in the determination of fungal diversity with emphasis on the efforts needed to increase our knowledge on these poorly understood organisms in the deep sea.

## 2. Brief Historical Perspective: From the Discovery of Marine Fungi to Current State-of-the-Art

The ascomycete *Phaeosphaeria typharum* was the first aquatic inhabiting fungus identified in 1849 [5]. Although it was found to be associated with the freshwater plant genus *Typha*, it was later classified as a facultative marine fungus. A few years later, the ascomycete *Sphaeria posidoniae* was found to be associated with the rhizomes of the sea grass *Posidonia aceanica* and it was classified as an obligate marine fungus [6]. During the following century, scarcely 50 species of fungi from the marine environment were described [7]. The interest for marine fungi increased soon after, when 25 new lignicolous marine fungal species were described at 1000 mbsl [8]. Since then, studies on marine fungi, and their symbiotic interactions with other organisms have been on the rise.

The definition of marine fungi has been fairly ambiguous, and the reason for this is straightforward: marine fungi have been described simply as fungi able to grow and reproduce under marine conditions, without taking into account their capacity to adapt to diverse marine environments such as salt marshes, hydrothermal zones, and the deep sea [9]. A less restrictive definition has been proposed, which describes a marine fungus as a fungus recovered recurrently from different marine environments, capable of growing and/or adapting metabolically to the marine biotic/abiotic conditions, and actively maintaining symbiotic interactions with other organisms [9,10]. Based on their ecology, marine fungi can be defined as obligate or facultative [11]. Obligate marine fungi are able to grow and sporulate exclusively in the marine environment, while facultative marine fungi are those from terrestrial or freshwater habitats that are able to grow and/or sporulate in the marine environment [7].

Marine fungi include species belonging to the phyla Ascomycota, Basidiomycota, Blastocladiomycota, Chytridiomycota, and Mucoromycota [12]. Currently, the count amounts to 1112 species, with the majority of them classified within Ascomycota (805 species), followed by Basidiomycota (21 species), Chytridiomycota (26 species), Mucoromycota (three species), Blastocladiomycota (one species), and 43 remaining unidentified fungal species classified as asexual morphs of filamentous fungi [13]. These numbers are rather conservative since more than 10,000 fungal species, except those categorized as unidentified fungal group, are estimated to exist in the marine environment [14]. The unidentified fungal group appears recurrently in studies where culture-independent approaches are used, and, most importantly, they constitute one of the most abundant groups in deep submarine canyons of the Mediterranean Sea and in the Gulf of Mexico [15,16].

**Figure 1:**
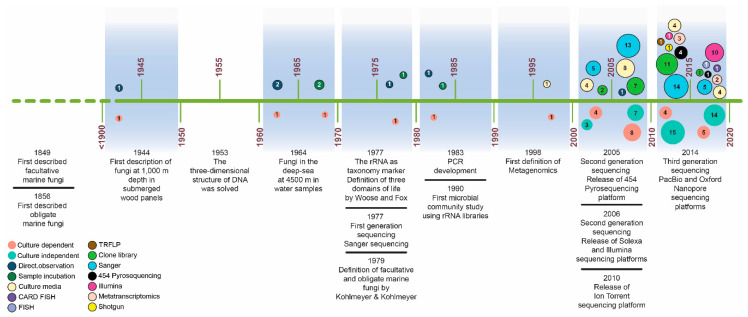
Timeline showing important studies of deep-sea fungi through culture-dependent and culture-independent approaches as well as the development of molecular techniques and sequencing platforms. Each circle represents the sum of the number of studies conducted every five years. Additionally, the different techniques used are also indicated.

Early studies that assessed the diversity of marine fungi in the deep sea used culture-dependent methods, clone libraries, or a combination of both techniques (Figure 1). In the second half of the 20^th^ century, few reports published on deep-sea fungi included the direct detection of fungi from submerged wood panels from 1000 to 5315 mbsl [8,17], the incubation of water samples and direct detection of fungi from zones at 4000 mbsl [18], and the barotolerance capacity of fungi isolated from deep-sea sediments at 965 mbsl [19] (Figure 1). In those studies, the fungi were identified by their morphology and growth characteristics. Once molecular tools were implemented, there has been a significant growing interest to evaluate marine fungal diversity (Figure 1). This has enabled the characterization of fungal communities in marine systems, providing unprecedented insight into spatial distribution patterns, their role in ecosystems and their potential response to natural and anthropogenic disturbances. During the first decade of the 21^st^ century, most of the studies combined culture-dependent approaches and the classical Sanger dideoxy chain-termination sequencing method of amplicon-based taxonomic barcodes, most commonly the rRNA 18S or internal transcribed spacer (ITS) 1 or 2 regions [4,20,21,22,23,24,25,26,27]. During that time, some of the first used high throughput sequencing (HTS) platforms (454-Pyrosequencing in 2005 and Illumina in 2007) were released. However, the application of NGS technologies for the study of fungi in the deep sea was not implemented extensively until the last decade [16,28,29,30,31,32,33,34,35,36,37,38,39,40,41].

**Figure 2:**
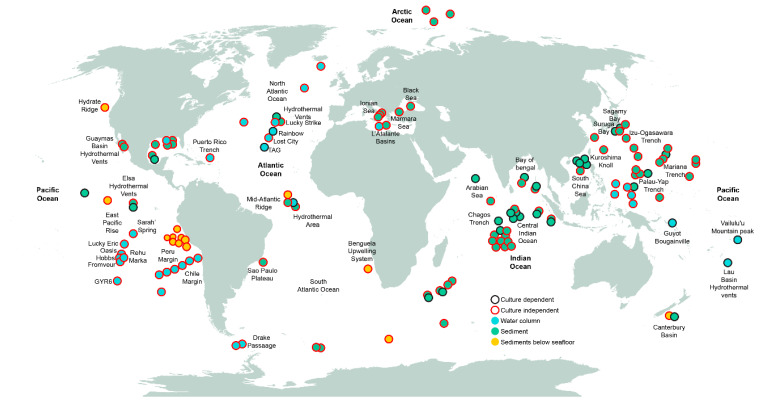
Worldwide locations where samples from deep-sea environments have been collected, and corresponding techniques used to assess fungal diversity. Data points are approximate according to geographic references in the publications.

We have reviewed the available published collection of studies (Table S1), in which culture-dependent techniques, culture-independent techniques, or a combination of both were used to analyze the diversity of fungi from deep-sea environments around the world (Figure 2). Most of the efforts to characterize fungi of the deep sea have been devoted to sediment samples from the Pacific and Indian Oceans. In addition, deep-sea water samples have been studied at depths ranging from 1000 to 6000 mbsl including the East Pacific Ocean, the Peru and Chile margins, and the North and Mid-Atlantic Ocean, mostly focusing on hydrothermal vents [20,25,26,42,43,44]. Culture-independent studies have provided greater area coverage than culture-dependent studies (Figure 2). The geographical bias derived from the higher sampling effort conducted in the Pacific and Indian Ocean may have an effect on the current knowledge of the fungal taxonomic diversity of the deep-sea environment. This hinders the construction of an accurate deep-sea distribution map with any degree of confidence. It may be necessary to build a database (or an inventory within a database) specific for deep-sea fungi (obtained by culture dependent and independent techniques) that contains records of taxonomic diversity including the under-sampled or non-sampled areas.

## 3. Omics Technologies: The Blooming Era of Marine Fungal Diversity

The emergence and development of HTS technologies [45] have greatly contributed to the advancement of metagenomics and metatranscriptomics methods. The combination of both methods is providing a wealth of information about the diversity and putative ecological roles of different microbial communities in environmental samples [46]. In the last decade, the growing interest in revealing the fungal diversity in all ecosystems including the marine ones, coupled with the development of HTS, has allowed for the sequencing of entire fungal communities [47].

The most commonly used HTS-based methods to assess fungal communities in a given environment use a targeted gene sequencing approach (amplicon-based) or a shotgun sequencing approach. The targeted gene sequencing approach is based on the amplification of a barcode gene, commonly the internal transcribed spacer (ITS) region for fungi. This method generates an enrichment of the fungal fraction of the community. Moreover, some primers could have a preferential affinity for some taxonomic groups of fungi, and PCR could favor the amplification of those groups [48]. In contrast, in the shotgun method, the data obtained corresponds to the entire microbial community (Bacteria, Archea, Fungi, Nematoda, etc.). Reconstruction of the fungal genomes from a total metagenome recovered from shotgun sequencing is challenging. Through shotgun sequencing, a genome profile of all members of the community (metagenome) is recovered by the assembly of short reads into larger sequences (contigs). However, the probability of recovering complete genomes for fungi by shotgun sequencing is lower than for bacteria. On one hand, the assembly of genomes depends largely on the abundance of the corresponding members of the microbial community in the sample, and fungi are generally found in less abundance than bacteria [49]. Second, the greater complexity and larger orders of magnitude of fungal genomes makes the assembly process more difficult. Finally, the large number of non-coding regions (introns) full of repeats in fungal genomes can cause arrangement errors and gaps during the assembling process [50].

Through shotgun metagenomics, aside from obtaining a taxonomic profile, we can assess the functional potential of the entire community by making inferences about metabolic functions. Even so, a drawback of studying fungi through shotgun sequencing is that fungal genome databases in contrast to bacterial genome databases are not as comprehensive, and this results in a poor fungal identification [51].

The continuous efforts to improve HTS technologies have focused on obtaining larger reads and diminishing errors. As a result, two HTS platforms have emerged: single-molecule real-time (SMRT) developed by Pacific Biosciences, and MinION developed by Oxford Nanopore Technologies, both generating longer reads (from 20 to 100 kb) than other sequencing platforms (i.e., Illumina) [52]. The use of these technologies can reduce the uncertainty on the marker to be used for fungal identification (ITS1 or ITS2) considering that the entire ITS region (typically 500–700 bp) could be sequenced, thus providing the advantage of higher taxonomic and phylogenetic resolution [52,53]. Furthermore, obtaining longer reads would make it easier to distinguish the genetic material of living cells from extracellular genetic material or material derived from damaged cells, consisting of fragments smaller than 200 bp [53,54].

Single-cell genomics (SCG) provide powerful tools to study uncultured fungi, but they are not yet widely implemented to explore the diversity and potential of fungi in deep-sea studies. SCG allows linking metabolic function to a single fungal species, without the necessity of culturing. This allows us to determine the probable role in the habitat of non-cultivable and low-abundant fungal species, which are commonly underrepresented or overlooked in metagenomics surveys [55]. Additionally, several phylogenetic markers from the individual cells can be detected, allowing a better identification of the species [56]. While this is a very promising approach, there are several technical difficulties associated with the first step of the process; in the case of fungi, the isolation of the single cells becomes a serious disadvantage given the different cellular morphologies they can present [57]. A recent study reported a pipeline for SCG applied to some uncultured species from Cryptomycota, Chytridiomycota, and Zoopagomycota. This new approach led to the detection of evolutionary patterns as well as metabolic deficiencies and gene expansions among the studied fungi [57]. Applying this approach to deep-sea fungi could help us to understand more about their evolutionary history and provide insights into unresolved evolutionary questions.

To the best of our understanding, no proteomics or metabolomics analyses have been reported for fungal communities of the deep-sea environment. Proteomics analysis allows for the assessment of community function, and has the advantage over metatranscriptomics that proteins have a greater lifetime than mRNA [58]. A tandem mass spectrometer (MS/MS) is used to obtain the protein profiles of the community [59], meaning that these profiles could provide insights as to whether there were active organisms at the moment when the sample was taken. Furthermore, the identified proteins could be related to a specific microbial group and could be linked to important metabolic pathways [58,59] as well as indicate differential expression over spatial and temporal scales. This may strongly suggest shifts in the microbial composition of the community [58]. Metametabolomics analyzes the metabolic products resulting from the microbial community, which could provide information on the interactions within a community in which a metabolite produced by an organism can be used by other organisms [58]. As with other omics techniques, proteomics and metabolomics face different challenges associated with sample manipulation and the identification and quantification of the obtained (protein or metabolite) profiles [59]. When some of these omics techniques are considered in the study of fungi in deep-sea environments, it will be important to note that fungi may have low-biomass representation in the community, meaning that the amount of sample has to be taken into account during sampling in order to obtain sufficient material for reliable protein and metabolomics profiles.

## 4. Out of the Dark: The Hidden Mycota of the Deep Sea and Their Physiological Characteristics

At first, it was suggested that the deep-sea environment does not harbor a high diversity of fungi. Nevertheless, with the increasing amount of data obtained with HTS-based approaches, this idea is no longer supported. There are substantial differences in the number of fungi recovered using culture-dependent or culture-independent techniques. For example, in two studies carried out in the East and Central Indian Ocean, a total of 39 cultivable fungi were obtained. In contrast, 91 fungal OTUs (Operational Taxonomic Units) were obtained using culture-independent methods [60,61]. Currently, examples of genera from all taxa have been identified in the deep sea. The majority of them belong to the phyla Ascomycota and Basidiomycota, although there is also a lower diversity and abundance that belong to the phyla Chytridiomycota, Neocallimastigomycota, and Glomeromycota (Table S2) [3,4,15,16,20,21,23,40,43,62,63].

**Figure 3:**
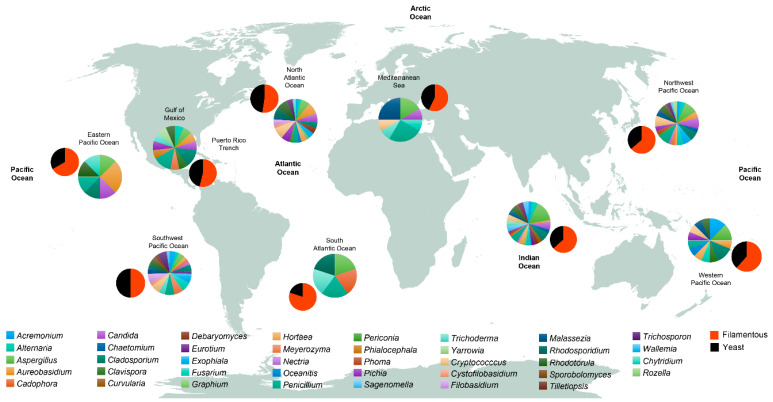
Worldwide distribution of fungal genera and fungal morphologies. Data in pie charts are from studies that reported the presence of fungi at the genus level. Only genera that were present in two or more studies were included. Studies that only reported fungi as a whole group were discarded. For further information, the reader is referred to Table S2. Smaller pie charts represent the proportion of the different fungal forms (Filamentous and Yeast).

From the analyzed studies examined in this review (Table S1) including both culture-dependent and independent methods, we have grouped the different sampled stations by their geographical location (Figure 3) and extracted the most reported genera. Within the Dikarya, we found that there is a preponderance of filamentous forms over yeast forms in the deep sea, although data from the Pacific Ocean’s Mariana Trench at a depth of more than 10,800 mbsl revealed that members of the red yeast *Rhodotorula* dominated the fungal community [3]. Worldwide, the most reported genera of filamentous fungi are *Penicillium*, *Aspergillus*, *Aureobasidium*, *Cladosporium*, *Trichoderma*, *Alternaria*, *Acremonium*, *Fusarium*, *Hortaea*, and *Exophiala*; while the most reported genera of yeast forms are *Rhodotorula*, *Candida*, *Malassezia*, *Cryptococcus*, *Pichia*, *Rhodosporidium*, and *Trichosporon* (Figure 3; Table S2). From the most reported genera, *Aspergillus*, *Penicillium*, *Aureobasidium*, *Cladosporium*, *Rhodotorula*, and *Candida* represent the conserved community of the deep-sea environment. Globally, the northwest and southwest Pacific Ocean, the north Atlantic Ocean, and the Indian Ocean have the highest number of reported genera in comparison with other zones such as the western and eastern Pacific Ocean and the south Atlantic Ocean (Figure 3). The wide ranging variability in the number of fungal genera in the different zones could be explained by the disparities in the sampling effort: while some zones are highly sampled (i.e., the Indian Ocean), some others, for example, the South Atlantic Ocean, have been poorly sampled (Figure 2). We lack information on detailed fungal diversity in the deep-sea environment from the Arctic Ocean, where the few sampled points that have been studied (Figure 2), have reported fungi as a general group, without specifying lower taxonomic ranks [42]. *Oceanitis* and *Abyssomyces* are two genera identified as obligate marine fungi that were isolated from submerged wood panels in the north Atlantic Ocean [17].

The presence of fungi in the deep-sea environment belonging to the Chytridiomycota and Glomeromycota, considered basal fungal lineages, indicates that the deep-sea sediments harbor ancient fungi. Significantly, the sequences recovered from different deep-sea sources belonging to Chytridiomycota, Cryptomycota, and other basal fungi are distant from their counterparts identified in terrestrial environments [64]. Some members of the Chytridiomycota and of the novel fungal groups encountered have been detected in deep-sea sediments of hydrothermal vents at more than 2500 m [23], methane cold-seeps at 1200 m [65], and submarine canyons at 1000 m [15]. It is important to highlight that these novel fungal groups have only been detected in studies where culture-independent techniques have been applied [64]. The novel deep-branching lineages described in deep-sea methane cold-seeps sediments [65] and in the Mariana Trench [4] could provide information about the origin and evolution of fungi and help resolve the ongoing debate on whether the large proportion of terrestrial/facultative fungal species detected in the deep sea [16,66] occur naturally in that environment or originate from terrestrial sources [67]. The rivers that flow into the ocean could be some of the contributing sources enriching the marine sediments with facultative fungi; this could explain why the fungal biomass from marine sediments is comparable to the fungal biomass from soils in terrestrial environments, which represents ~0.5 to 4% of the entire microbial biomass [68]. Comprehensive analysis of genomic data, specifically of some genes of importance in the evolution of fungi, will provide a better understanding on how fungi have adapted to these two contrasting environments [69]. Fungi of the strictly anaerobic phylum Neocallimastigomycota live in animal guts and are rarely reported in the deep-sea environment. Their detection in environmental samples [16,70] has been attributed to possible remains of marine mammal corpses or to the presence of dormant spores.

Subseafloor sediments also contain an important sum of fungi that could contribute to understanding the ecophysiological adaptation of fungi in marine environments. Depending on their geographical location, deep-sea sedimentation rates vary from 0.1 m/10^6^ years to 30 m/10^6^ [71]. This means that subseafloor sediments (i.e., the Canterbury basin at more than 1927.5 mbsf [31,72]) could be millions of years old, and the presence of ancient fungi would be expected. However, as in the seafloor, the subseafloor sediments studied were dominated by commonly found genera of Dikarya fungi (*Acremonium*, *Aspergillus*, *Cladosporium*, *Fusarium*, *Penicillium*, *Meyerozyma*, *Rhodotorula*, and *Cryptococcus*), while other fungal groups were found in low abundance. Genetic analyses of subseafloor fungi isolated from the Canterbury Basin identified differences in their machinery for the production of secondary metabolites and suggested a potential loss or gain of genes in the different species as a result of their physiological adaptation to the conditions of the corresponding subseafloor layer, from which they were recovered [72]. Fungi found in subseafloor sediments have also been proven to prevail in a dormant state for long periods. A comparison among metatranscriptomes from terrestrial and subseafloor fungi revealed a high overexpression of genes encoding members of the SOR/SNZ protein family involved in cellular maintenance, and proteins associated with autophagy [73]. This strategy of cellular maintenance represents a strategy for survival under nutrient-poor conditions as the ones found in deep-sea environments.

The growth and activity of fungi in the deep sea depend on the byproduct of the biological pump, which falls from upper layers of the water column to the bottom; the labile compounds are degraded first and the resulting recalcitrant compounds (i.e., humic components) remain accumulated and buried in the sediment until degraded [74]. It is in the degradation of organic material, labile or recalcitrant, where fungi could have an active role, as it occurs in the terrestrial environments [75]. However, the dynamics of fungal communities in this type of ecosystem is still poorly understood and unexplored.

Environmental genomics (EG) allows making inferences about fungal activity from taxonomic composition. In addition, EG seeks to correlate the response of the organisms to their inhabiting environment by incorporating meta-omics analyses focused on the transcriptome, proteome, and metabolome components (from individuals to communities) at different spatio-temporal scales [76,77]. Applying EG could guide us to resolve not only the gaps in fungal evolution, but also the molecular mechanisms involved in the capacity of fungi to inhabit the deep sea and their gene-expression responses to variations in this extreme environment [77].

Unfortunately, studying fungi in the deep-sea environment through the point of view of EG is still in its infancy. In the last decade, only a handful of studies have been dedicated to implement the metatranscriptomic fungal analysis of deep-sea sediment samples (Figure 1). Most of these studies were carried out in the Peru Margin (Figure 2), where samples were obtained through the Ocean Drilling Program, and different depths below the seafloor (159 to 345 mbsf) were reached. These studies of the Peru Margin sediments identified fungi to be present and active in all sampled depths. Transcripts related to carbohydrate, amino acid, lipid metabolism, and hydrolases were identified, suggesting an active participation of fungi on different substrate degradation processes [34,35,36]. Furthermore, transcripts related to hyphal growth and ergosterol biosynthesis were detected [36].

Marine fungi in the deep sea may have potential physiological capacities associated with the extreme characteristics of the habitat. The deep-sea biosphere is characterized by exhibiting high hydrostatic pressure (1 bar/10 m), low temperatures (2–4 °C) or extremely high (>400 °C) in the case of hydrothermal vents [4], high salinity concentrations (240 ppt) reaching up to 500 ppt in deep hypersaline basins [78,79], and the absence of photosynthetic processes due to the lack of light [4,80]. Among the abiotic conditions that fungi can face in the deep-sea environment, their capacity to withstand high hydrostatic pressures is noteworthy. Most of the early studies did not test the barotolerance of fungi isolated from deep-sea sediments [7]. *Aspergillus ustus* and *Graphium* sp., isolated from the Indian Ocean, were tested for barotolerance, and presented conidia germination at a pressure of 100 bar (10 MPa) [19]. *Aspergillus sydowii* can grow and sporulate at 500 bar (50 MPa), which represents the pressure found at 5000 mbsl [62,81]. Moreover, facultative fungi capable of colonizing the deep sea can modify the composition and thereby the fluidity of their plasma membrane to regulate the pressure [82]. This physiological adaptation has been confirmed by the detection of high levels of transcripts involved in the biosynthesis of ergosterol, suggesting that marine fungi could undergo changes in their membrane composition to endure high hydrostatic pressures [36]. *Saccharomyces cerevisiae* grown under a high hydrostatic pressure of 2000 bar (200 MPa) resulted in the upregulation of the *OLE1* gene, which is involved in the synthesis of fatty acids [82]. In addition, another important characteristic for the survival and growth of fungi in deep-sea environments is their ability to grow under a wide range of salt concentrations. Even when marine fungi are able to tolerate high salt concentrations, they are not commonly halophilic [78]. The term halotolerant (or halophytic) refers to the capacity of growing with salt without being a growth requirement. In contrast, the term halophilic refers to the need of a certain salt concentration for optimal growth, ranging from moderate (0.6 M of NaCl) to extreme (more than 5 M of NaCl) concentrations [83,84]. Some species from the genera *Penicillium*, *Cladosporium*, and *Aspergillus* isolated from 5700 mbsl are able to sporulate in a range of 1.7 to 34 ppt, proving to be halotolerant organisms [63]. A new species from the genera *Candida*, isolated from a deep-sea hydrothermal vent, has been proven to be halophilic, since it was able to grow only under a salinity concentration of 30 ppt [83]. In deep-sea hypersaline basins (salinity between 70 and 172 ppt) located in the Eastern Mediterranean Sea that harbored Ascomycota and Basidiomycota [85], a metatranscriptomic analysis revealed that the majority of the detected rRNA signatures were related to the genera *Aspergillus* and *Penicillium* [33]. The genus *Hortaea* is commonly detected from deep-sea environments [22,60,61,66,83,86,87], specifically the species *H. werneckii*, a black yeast that has been classified as halotolerant in Mediterranean deep-sea waters [88] and as halophilic in deep-sea hydrothermal vents [83]. Marine-derived *Penicillium* and *Aspergillus* species are an excellent and widely used source for many useful bioactive secondary metabolites [89,90]. A total of 106 novel bioactive compounds have been identified from *Penicillium* and *Aspergillus* species isolated from the deep sea [91]. Some of the compounds obtained from *Penicillium* species isolated from deep-sea sediments in Japan have been characterized as compounds with high cytotoxicity effects on different cancer cell lines [91,92]. Some other deep-sea fungal species belonging to the *Simplicillium*, *Acaromyces*, and *Engyodontium* genera have proven to be a rich source of secondary metabolites with cytotoxic activity against different cell lines [93]. The successful antimicrobial activity of six novel alkaloid-bioactive compounds isolated from *Aspergillus* species has been tested against some fungal and bacterial pathogens such as *Candida albicans*, *Staphylococcus aureus*, *Pseudomonas aeruginosa*, and *Klebsiella pneumonia* [91]. It is now well established that the inordinate number of diverse fungi in the deep sea can be regarded as a rich source of novel bioactive compounds including anticancer, antibacterial, antiviral, antifungal, antioxidant, and non-toxic antifouling compounds. The different omic techniques will help to describe many more novel compounds [79,91,94,95].

## 5. The Difficult Task of Assessing Marine Fungal Diversity of the Deep-Sea Sediments

The use of culture-independent techniques has allowed us to extend our knowledge about both uncultured fungi and cryptic fungi (fungi with similar morphology that cannot be differentiated by microscopic techniques) present in the marine environment [96]. In addition, it has enabled the discovery of new fungal groups such as the DSF-Group1 (Deep Sea Fungi-Group 1), closely related to the phylum Ascomycota and is suggested to represent a globally distributed group of fungi related to deep-sea anaerobic environments [4]. The description of the DSF-Group1 in several deep-sea samples has helped reveal unknown fungal diversity [56]. Through HTS platforms, a new group of fungi has been identified that is commonly referred to as “unclassified” or “unidentified” fungi. Often the unidentified fungal group represents a high proportion of the analyzed data, suggesting that the deep sea harbors a largely unknown fungal community, which could include members of potential biotechnological importance [15,16].

As mentioned in Section 3, the most widely used barcodes for fungal identification are the ITS regions (ITS1 and ITS2) [97,98,99]. These regions show a great variability in size and sequence among different taxa, allowing identification to genus and species levels [22,99,100,101,102]. In addition, the number of ITS copies per fungal cell is typically more than 250, which makes this region an ideal target gene for fungal identification, especially in studies where a low concentration of environmental DNA (eDNA) is recovered from the environment to be studied [47]. Hence, the use of these two variable regions (ITS1 and ITS2) has contributed greatly to the progress of fungal diversity research. The ITS1 region has more intra-strain sequence heterogeneity than the ITS2 region, which is advantageous to set apart different strains of the same species. At the same time, the different copies of the ITS1 region within a fungal strain may vary in size (around 13% between repeats), and this hinders the identification of novel species [103]. Currently, there is a wide range of universal and specific oligonucleotides for each taxon, which allows the amplification of the fungal ITS regions as well as the amplification of the small subunit (SSU) 18S and the large subunit (LSU) 28S rRNA. The SSU has a low evolution rate in comparison with the ITS region, which means a lower variation in the identification of taxa, making it suitable for the identification of fungi from phyla to family level [104]. While the LSU has been suggested to perform similarly to the ITS region for fungal identification at the genus level, the ITS region allows for identification at the species level due to sequence variability. Still, the LSU performed better for fungal phylogeny and taxonomic identification [105]. Overall, the combined use of several primers for different regions is desirable to improve the accuracy of fungal taxonomic identification [99].

## 6. Current Challenges

The development of HTS has allowed us to obtain large datasets and represents a more cost-efficient alternative to conventional techniques [106]. Massive sequencing technology has permitted the study of model and non-model organisms, contributing to the advancement of disciplines such as microbial ecology and evolution [107]. HTS has enabled sizeable global analysis of microbial communities including fungi associated with plant systems (mycorrhizae, endophytes, and pathogens), free-living saprotrophs, and fungi from extreme environments [48]. Nonetheless, studying fungal communities (identification and quantification of species) must take into account different technical aspects to obtain reliable data, formulate subsequent conclusions, and carry out comparative analyses between different sets of data. Some initiatives such as the Earth Microbiome Project (EMP) have offered clear instructions for the analysis of microbial communities in all types of environmental samples including sediments. Detailed protocols to extract genomic DNA, and perform 16S, 18S, and ITS1 amplicon sequencing libraries have been shared on their website [108]. This project has the aim to perform meta-analyses in order to identify global patterns of microbial communities and link these results with physicochemical parameters. One of the problems when trying to follow some standardized protocols to study fungal diversity such as the proposed by the EMP is that there is no consensus as to which molecular marker (i.e., entire ITS region, ITS1, and ITS2) must be used for fungal identification. The Illumina ITS-based amplicon protocol from EMP focuses on the amplification of the ITS1 region, even if it is not always the choice of the researcher. Another limitation is the resources available at different labs. Some of the standardized protocols use automatized equipment, for example, to purify DNA/RNA, which could be expensive and not accessible for some laboratories.

Another important aspect to consider is the recovery of possible contaminant species in the samples. It is crucial to have sequence representation from the possible contaminants through the handling and processing of the samples. Several controls should be included during sample collection, when the samples are prepared for preservation, during DNA/RNA extraction, and PCR. In culture-dependent techniques, open petri dishes containing different culture media are exposed in the same working space where the samples are prepared; for culture-independent techniques, this kind of control should also be included, especially in fungal labs with shared equipment. To obtain these controls, tubes with water or DNA preservation buffer should be kept open during all the sample processing steps and sequenced along with the samples [16].

The importance of these controls relies on the need to diminish the possible enrichment and presence of terrestrial species sequences in the collected samples, since a high abundance and diversity of facultative fungi has been detected in the deep-sea environment as discussed above. How to handle the contaminant sequences present in the controls will depend on the representation of these sequences in the samples, and since there is no consensus so far on how these sequences should be handled, different strategies could be applied: (1) delete all the sequences corresponding to the fungal OTUs present on the contaminant controls, or (2) eliminate a proportion of the sequences equal to the contaminant sequences present in the controls [109].

Additionally, it is highly recommended that a mock community control be included. This would first allow the evaluation of the quality of the sequencing, the fidelity of the analysis, establishment of the criteria for the correct determination of fungal richness, and the identification of the preferred database for the known sequences present in the Mock community [48,110]. This would help to improve the taxonomic classification of the obtained OTUs. The taxonomic classification may be highly dependent on the database used, and at the moment, there is no such specific database with enough information on marine fungi. The UNITE database [51] has been the one most widely used, since it contains a total of 2,480,043 ITS sequences and is a curated database, but there is no representation of marine fungal sequences. The main reason for the lack of representation of marine fungi in the databases is that not all studies deposit the data in a public repository. There are some online resources for marine fungi; one of them is the taxonomic database Marine Fungi (http://www.marinefungi.org/), which is focused on taxonomic information from fungi documented from submerged material, water, mangrove, and deep-sea sediments [12]. It contains data for a total of 1689 species, but it does not include genomic information. Another resource that can be used is the World record of marine species database (WoRMS) [111] with a total of 2439 fungal records, but no genomic comparisons can be made in the database. Similarly, regarding what occurs when mining the databases for fungal identification, there are some limitations when processing transcriptomic data. There is a low similarity of the transcripts assigned to a specific function with genes from public databases because of the shortage of fungal reference genomes derived from the marine environment [79]. Initiatives such as the 1000 Fungal Genomes Project, supported by the Joint Genome Institute, could be a great contribution if contemplating marine fungi representation.

## 7. Final Remarks

It has taken about 150 years (from 1849 to 2000) to boost marine fungal research in the deep sea and to publish the corresponding findings. Deep-sea fungi are still an unknown fraction of the marine environment. Greater coordinated efforts are needed to expand our current knowledge on their diversity, their activity, and their ability to adapt to the deep-sea conditions or in the case of obligate marine fungi, to understand how they actively thrive in this harsh marine environment.

Current and future studies on deep-sea fungi should make available to the community the obtained datasets, so that comparative analyses including the biogeography, composition, and abundance of fungi in this environment can be attained. Future marine mycology research should also focus on the commonly overlooked “unidentified” or “unclassified” sequences in the deep-sea fungal datasets, which aside from being of great biotechnological potential, could represent key fungal species for evolution theories about the origin of marine fungi.

## Supplementary Materials

The following are available online at https://www.mdpi.com/2075-1729/10/11/292/s1, Table S1: Database of studies of fungi in the deep-sea environment. Table S2: Fungal taxa found in the deep-sea environment.
